# Haplotype analysis of *XRCC2* gene polymorphisms and association with increased risk of head and neck cancer

**DOI:** 10.1038/s41598-017-13461-6

**Published:** 2017-10-16

**Authors:** Soma Saeed, Ishrat Mahjabeen, Romana Sarwar, Kashif Bashir, Mahmood Akhtar Kayani

**Affiliations:** Cancer Genetics and Epigenetics Lab, Department of Biosciences, COMSATS Institute of Information Technology (CIIT), Park Road, Chak shazad Islamabad, Pakistan

## Abstract

We aimed to investigate the effect of hotspot variations of *XRCC2* gene on the risk of head and neck cancer (HNC) in 400 patients and 400 controls. Five polymorphisms of *XRCC2* gene G4234C (rs3218384), G4088T (rs3218373), G3063A (rs2040639), R188H (rs3218536) and rs7802034 were analyzed using Allele- specific polymerase chain reaction (ARMS-PCR) followed by sequence analysis. For rs3218373, the GG genotype indicated a statistically significant 3-fold increased risk of HNC (P < 0.001) after multivariate adjustment. For rs7802034, the GG genotype suggested statistically significant 2-fold increased risk of HNC (P < 0.001). For SNP of rs3218536, the AA genotype indicated a significant 3-fold increased risk of HNC (*P* < 0.001). Additionally, haplotype analysis revealed that TACAG, TGGAG, TACGG and TAGGA haplotypes of *XRCC2* polymorphisms are associated with HNC risk. Two SNPs in *XRCC2* (rs2040639 and rs3218384) were found increased in strong linkage disequilibrium. Furthermore, joint effect model showed 20 fold (OR = 19.89; 95% CI = 2.65–149.36, P = 0.003) increased HNC risk in patients carrying four homozygous risk alleles of selected polymorphisms. These results show that allele distributions and genotypes of *XRCC2* SNPs are significantly associated with increased HNC risk and could be a genetic adjuster for the said disease.

## Introduction

Various damaging agents such as chemicals, radiations and some endogenous elements affect DNA integrity which ultimately result in single strand breaks (SSBs). Unrepaired SSBs may lead to double strand breaks (DSBs) during the S phase of the cell cycle^1^. Accumulation of these unrepaired DSBs can cause cell death and initiate malignancies^2^. There are several mechanisms which repair these DSB. Homologous recombination repair (HRR) is the key pathway for this DNA repair, functioning in S phase of somatic mammalian cell cycle^2^. Defective HRR has been reported to be closely related to different human cancers^3^. A wide range of crucial molecules have been identified to participate in HRR process such as *RAD51* paralogs (*RAD51B, RAD51C, RAD51D*, *XRCC2*, *XRCC3*) and can serve as central proteins during HRR process^4,5^. X-ray repair cross complementing group 2 (*XRCC2*) gene, *XRCC2* protein, together with other proteins of *RAD51* ^6^, forms a complex which plays a critical role in chromosomal segregation and apoptotic response to DSBs^7^. This crucial function of the *XRCC2* protein for the HRR process has been demonstrated in earlier studies where over 100 fold HRR reduction in *XRCC2* deficient hamster cells was observed compared to parental cells^8^.

Many earlier studies have found that single nucleotide polymorphisms (SNPs) in the DNA repair gene might modify DNA repair capacity and subsequently influence the susceptibility of cancer^9^. Common genetic variants in *XRCC2*, particularly a coding SNP in exon 3 (R188H, dbSNP ID rs3218536), have been identified as potential cancer susceptibility loci recently, though the association results were controversial^6,10^. The *XRCC2* R188H polymorphism has been proposed to be a genetic modifier for smoking related pancreatic cancer^10^, pharyngeal cancer^11^, oral cancer^12^ and ovarian cancer^13^, though validation studies could not provide confirmation^14,15^. Some of the studies have also implicated *XRCC2* R188H in breast cancer^16–19^, however, the Breast Cancer Association Consortium^20^ and other subsequent studies found no association between R188H and breast cancer risk^21,22^, or evidence of a modest protective association^23,24^.

Since *XRCC2* genomic sequence is highly polymorphic, it is of interest to identify genetic defects which have a functional potential to affect the final repairing efficiency of XRCC2, and subsequently the development of head and neck cancer. On the basis of these observations, it was planned to study the role of *XRCC2* gene as a candidate involved in the underlying cause of head and neck carcinogenesis.

## Materials and Methods

### Study subjects and ethical approval

The subjects included in this study consisted of 400 diagnosed head and neck cancer patients and an equal number of age- and sex-matched controls. These subjects were collected during 2011 to 2015. The diagnosis of the head and neck cancer patients was made histologically at Nuclear Oncology Radiation Institute (NORI) Islamabad and Pakistan institute of medicine Sciences (PIMS). Controls were selected from individuals receiving routine medical examinations in these hospitals. The selection procedure for patients included confirmed histological diagnosis of HNC, no preoperative therapy and availability of complete follow-up data. The inclusion criterion for the controls was age and sex matched healthy individuals with absence of prior history of cancerous or precancerous lesions. Patients and controls suffering from any other familial disease (diabetes, blood pressure, and cardiovascular, renal, or hepatic impairment) were excluded from this study. A written informed consent was obtained from all subjects. Additionally, a structured questionnaire, including information about demographic factors, smoking habits and dietary habits was also used to interview all subjects who provided written informed consent. Peripheral blood samples were collected from all study subjects. This study was approved by the institutional Ethical Review Boards of COMSATS Institute of Information Technology, Islamabad and both collaborating hospitals. Additionally, all experiments performed were in accordance with relevant guidelines and regulations.

### DNA extraction

Approximately 3–4 ml blood sample was collected in vacutainer tubes from enrolled subjects in this study. DNA was extracted from whole blood by Phenol chloroform method with some modifications^25^. The extracted DNA was quantified by 2% ethidium bromide gels and spectrophotometrically using Nano Drop (Thermoscientifiv, USA) and stored at −20 °C until used.

### SNPs selection

Five functional polymorphisms in DNA repair gene *XRCC2* were selected using a set of web-based SNP selection tools (http://snpinfo.niehs.nih.gov/snpinfo/snpfunc.htm). Potential functional SNPs were included to meet the following criteria: (1) Validated SNPs with minor allele frequency > 5% in the Asian population; (2) SNPs present in the promoter of *XRCC2* gene such as G4234C (rs3218384), G4088T (rs3218373) G3063A (rs2040639); (3) SNPs present in exonic region of *XRCC2* gene such as R188H (rs3218536); (4) SNPs present in intronic region of *XRCC2* gene such as (rs7802034).

### Genotyping

Genotyping was performed by Allele- specific polymerase chain reaction (ARMS-PCR). Primers for PCR amplification were designed by WASP (web based allele specific primer designing tool)^26^. Primers specific for each polymorphism are given in Supplemetary Table S1. PCR reaction was carried out in a reaction volume of 10 µl containing 50–100 ng genomic DNA, 100 µ M of each primers and Solis BioDyne master mix. Thermal cycling protocol used was: 94 °C for 30 sec, optimized annealing temperature for 45 sec, 72 °C for 1 min and final extension for 7 minutes. PCR products were visualized on a 2% agarose gel electrophoresis (100 V, 300 A for 45 min). Products by the presence or absence of bands specific for wild or mutant primers, in each well, were evaluated using UV trans illuminator (Gel Doc BioRad, USA). Internal control β-Actin was used in each reaction as a positive control for PCR. PCR products of thirty patients with homozygous wild, homozygous mutant and heterozygous mutant genotype were further confirmed by sequence analysis. Thirty control (normal) PCR product with different genotypic patterns were also sequenced along with cancerous samples to compare the sequencing results. DNA sequencing was carried out by MC lab (USA) by automated fluorescent sequencing to verify both nucleotide sequence and presence of specific SNPs. Results of DNA sequencing were analyzed using BioEdit software version7.0.5.

### Statistical analyses

Statistical analysis was performed using GraphPad prism software v 6.0. The chi-squared test and one sample t-test was performed to assess difference of collected data of age, gender, family history, smoking status, histological type of HNC and different treatment modalities for HNC between the control and case group. Hardy-Weinberg equilibrium test was used to compare the actual genotypes with the expected number based on Hardy-Weinberg equilibrium theory (p = allele frequency, q = 1-p, p2 + q2 = 1) in controls. The difference in allele frequencies and genotypes between the control and case group was analyzed by Chi-squared tests. Logistic regression, with the adjustment of age and gender, was applied to calculate the odds ratios (ORs) and 95% confidence intervals (CIs). Three logistic regression models (additive, dominant, and recessive) were also used to analyze the SNPs. For SNP-SNP interactions, we used a adjusted logistic regression model to estimate the multiplicative interaction effect of the SNPs. P < 0.05 was considered to be statistically significant.

Haplotypes were generated from the genotyped data. The linkage disequilibrium (LD) and haplotype analysis were performed using Haploview 4.2, which uses the expectation maximization (EM) algorithm. Bonferroni correction was used to account for multiple testing and a two-tailed p value < 0.01 (=0.05/5 SNPs) was considered statistically significant.

## Results

### Case-Control Study

400 head and neck cancer patients and 400 control subjects were tested for five selected SNPs of *XRCC2* gene (rs3218373, rs2040639, rs3218384, rs7802034 and rs3218536). Demographic data of these head and neck cancer patients and control individuals is given in Table 1.

**Table 1:** Frequency distribution analysis of selected demographic and risk factors in head and neck cancer cases and controls.

*Variables*	*Cases* (*N* = *400*)	*Controls* (*N* = *400*)	*OR* (*95%CI*)	*P-value*
**Age (years)**				
Median (Range)	45 (17–68)	45 (22–65)		
**Gender**				
Males	243 (60.7%)	272 (68%)		
Females	157 (39.3%)	128 (32%)		0.06^a^
*Age*				
≤45	289 (72.3%)	251 (62.7%)		
>45	111 (27.7%)	149 (37.3%)		0.09^a^
**Family History of Cancer**				
Yes	153 (38.3%)	21 (5.3%)	11.17(6.89 to 18.1)	<0.0001^b^
No	247 (61.7%)	379 (94.7%)		
**Smoking History (cigarette, paan, bidi, betel quid, moist sniff)**				
Smokers	259 (64.7%)	227 (56.7%)	1.39 (1.05 to 1.86)	0.02^b^
Non-Smokers	141 (35.3%)	173 (43.3%)		
***Types of head and neck cancer***				
Oral Cavity	182(45.5%)	—		
Nasal Cavity	88 (22%)	—		0.03^a^
Pharynx	78 (19.5%)	—		
*Larynx*	52 (13%)	—		
**Types of treatment**				
Radiotherapy	88 (22%)	—		
Chemotherapy	94 (23.5%)	—		0.09^a^
Surgery	218 (54.5%)	—		

Abbreviations: N, number of samples; OR, odds ratio (crude); CI, confidence interval; level of significance p-value ≤ 0.05 calculated by one samples t-test^a^ and χ² -test^b^.

### Distribution of the *XRCC2* SNPs in head and neck cancer

The distribution of the genotypes and the allele frequencies of all of the studied polymorphisms are shown in Table 2. A significant association was observed between *XRCC2* and head and neck cancer. In case of first selected SNP (rs3218373) of *XRCC2* gene, frequency of heterozygous mutant (TG) and homozygous mutant (GG) genotypes was observed significantly higher in HNC patients compared to healthy controls (OR: 2.14, 95% CI: 1.47 to 3.10, P = 0.0001; OR: 2.58, 95% CI: 1.77 to 3.74, P < 0.0001 respectively). Frequency of G allele of respective polymorphism (rs3218373) was also found statistically higher in the patient group (OR: 2.73, 95% CI: 2.18 to 3.4, P < 0.0001). Genotyping of second selected SNP (rs2040639) of *XRCC2* showed that the frequency of G allele of respective SNP was significantly higher in the control group (OR: 0.64, 95% CI: 0.49 to 0.81, P = 0.0003) compared to patients as shown in Table 1.

**Table 2:** Distribution of five selected SNPs in *XRCC2* gene in head and neck cancer.

Genotype/Allele	Cases n (%)	Controls n (%)	OR (95% CI)	P- value
**rs3218373**				
TT	197 (49.2%)	299 (74.7%)	1	1
TG	97 (24.3%)	52 (13%)	2.14 (1.47 to 3.10)	**P** < **0.0001**
GG	106 (26.5%)	49 (12.3%)	2.58 (1.77 to 3.74)	**P** < **0.0001**
T allele frequency	491 (61.3%)	650 (8%)	1	1
G allele frequency	309 (38.7%)	150 (19%)	2.73 (2.18 to 3.4)	**P** < **0.0001**
**rs2040639**				
AA	301 (75.3%)	259 (64.7%)	1	1
AG	60 (15%)	85 (21.3%)	0.65 (0.45 to 0.94)	P = 0.0223
GG	39 (9.7%)	56 (14%)	0.66 (0.42 to 1.02)	P = 0.0645
A allele frequency	662 (82.7%)	603 (75%)	1	1
G allele frequency	138 (17.3%)	197 (25%)	0.64(0.49–0.81)	**P = 0.0003**
**rs3218384**				
GG	331 (82.7%)	269 (67.3%)	1	1
GC	48 (12%)	79 (19.7%)	0.55 (0.37 to 0.81)	P = 0.003
CC	21 (5.3%)	52 (13%)	0.37 (0.21 to 0.62)	**P = 0.0002**
G allele frequency	710 (88.7%)	617 (77%)	1	1
C allele frequency	90 (11.3%)	183 (23%)	0.42 (0.32 to 0.56)	**P** < **0.0001**
**rs7802034**				
AA	232 (58%)	199 (49.7%)	1	1
AG	101 (25.3%)	170 (42.5%)	0.45 (0.33 to 0.61)	**P** < **0.0001**
GG	67 (16.7%)	31 (7.8%)	2.39 (1.52 to 3.75)	**P** < **0.0001**
A allele frequency	565 (70.6%)	568 (71%)	1	1
G allele frequency	235 (29.4%)	232 (29%)	0.98 (0.79 to 1.22)	P = 0.699
**rs3218536**				
GG	240 (60%)	336 (84%)	1	1
GA	64 (16%)	26 (6.5%)	2.73 (1.69 to 4.42)	**P** < **0.0001**
AA	96 (24%)	38 (9.5%)	3.00 (2.00 to 4.51)	**P** < **0.0001**
G allele frequency	544 (68%)	698 (87%)	1	1
A allele frequency	256 (32%)	102 (13%)	3.22 (2.49 to 4.16)	**P** < **0.0001**

Abbreviations: n, Number of samples; OR, odds ratio; CI, confidence interval; p-value ≤ 0.05 considered as statistically significant. P-values in bold have still maintained their significance after Bonferroni correction (0.05/25 = 0.002). ORs were adjusted for age, sex and smoking status of cancer in logistic regression model.

Genotyping of third selected SNP (rs3218384) of *XRCC2*1 revealed that the frequencies of heterozygous mutant (GC) (OR: 0.55, 95% CI: 0.37 to 0.81, P = 0.003) and homozygous mutant (CC) (OR: 0.37, 95% CI: 0.21 to 0.62, P = 0.0002) genotype along with C allele frequency (OR: 0.42, 95% CI: 0.32 to 0.56, P < 0.0001) were significantly higher in the control group compared to patients. In case of fourth selected SNP (rs7802034), frequency of heterozygous mutant (AG) genotype was significantly higher in control group compared to patient group (OR: 0.45, 95% CI: 0.33 to 0.61, P < 0.0001). However, the frequency of homozygous mutant (GG) genotype was statistically significantly higher in patients compared to control group (OR: 2.39, 95% CI: 1.52 to 3.75, P < 0.0001). Genotyping of fifth SNP (rs3218536) showed that frequency of heterozygous mutant (GA) (OR: 2.73, 95% CI: 1.69 to 4.42, P < 0.0001) and homozygous mutant (AA) genotypes (OR: 3.00, 95% CI: 2.00 to 4.51, P < 0.0001) was significantly higher in patients compared with the controls. Moreover, the frequency of the A allele of respective SNP (rs3218536) was also statistically higher in the patients (OR: 3.22, 95% CI: 2.49 to 4.19, P < 0.0001) as shown in Table 1. Additionally hospital specific analysis (i.e. cases from Hospital 1 only; and then cases from Hospital 2 only) was also performed and no significant difference in frequency of selected polymorphisms was observed in patients from hospital 1 compared to patients from hospital 2 (Supplementary Table S2).

In genetic association studies, statistical power to detect disease susceptibility loci depended on the genetic models tested. Therefore, the genotype frequencies were further analyzed by three genetic models: additive, dominant, and recessive model. For rs3218373, a significant association between this polymorphism and increased HNC risk was found in dominant model (OR = 3.05, 95% CI = 2.26–4.11, P < 0.0001), recessive model (OR = 2.58, 95% CI = 1.77–3.74, P < 0.0001) and additive model (OR = 2.73, 95% CI = 2.20–3.48, P < 0.0001). Similarly, an increased risk of HNC risk was also found in recessive model (OR = 2.39, 95% CI = 1.52–3.75, P = 0.0001) of polymorphism rs7802034. Moreover, significant positive correlations between rs3218536 and HNC risk were also identified in dominant (OR: 3.50, 95% CI = 2.51–4.88, P < 0.0001), recessive (OR: 3.00, 95% CI = 2.00–4.51, P < 0.0001) and additive model (OR: 3.22, 95% CI = 2.49–4.15, P < 0.0001) as shown in Table 3.

**Table 3:** Analysis of the five selected SNPs based on three genetic models.

XRCC2 Genotype/Allele	Model	OR (95% CI)	p- value
**rs3218373**			
TT vs TG + GG	Dominant	3.05(2.26–4.11)	<**0.0001**
G/G vs TT + TG	Recessive	2.58(1.77–3.74)	<**0.0001**
G vs T	Additive	2.73 (2.20–3.48)	<**0.0001**
**rs2040639**			
AA vs AG + GG	Dominant	0.60(0.44–0.82)	**0.001**
GG vs AA + AG	Recessive	0.66(0.42–1.02)	0.06
G vs A	Additive	0.64(0.49–0.81)	**0.0003**
**rs3218384**			
GG vs GC + CC	Dominant	0.42(0.31–5.97)	<**0.0001**
CC vs GG + GC	Recessive	0.37(0.21–0.62)	**0.0002**
C vs G	Additive	0.43(0.32–0.56)	<**0.0001**
**rs7802034**			
AA vs AG + GG	Dominant	1.39(1.05–1.84)	0.02
GG vs AA + AG	Recessive	2.39(1.52–3.75)	<**0.0001**
G vs A	Additive	1.02(0.82–1.26)	0.87
**rs3218536**			
GG vs GA + AA	Dominant	3.50(2.51–4.88)	<**0.0001**
AA vs GA + GG	Recessive	3.00(2.00–4.51)	<**0.0001**
A vs G	Additive	3.22(2.49–4.15)	<**0.0001**

Abbreviations: OR, odds ratio; CI, confidence interval; p-value ≤ 0.05 considered as statistically significant. P-values in bold have still maintained their significance after Bonferroni correction (0.05/15 = 0.003). ORs were adjusted for age, sex and smoking status of study cohort in logistic regression model.

### Association of the *XRCC2* SNPs with different histological subtype and smoking status of HNC patients

Frequencies of selected polymorphisms were calculated in different histological subtypes of head and neck cancer such as oral, nasal, pharyngeal and laryngeal carcinoma. In case of oral carcinoma, mutant genotype of polymorphism rs3218373 (OR = 1.94; 95% CI; 1.24–3.04; P = 0.003), rs2040639 (OR = 0.31; 95% CI; 0.16–0.59; P = 0.0004), rs3218384 (OR = 0.07; 95% CI; 0.01–0.30; P = 0.0003) and rs3218536 (OR = 1.93; 95% CI; 1.16–3.22; P = 0.01) were observed significantly associated with oral carcinoma. In case of nasal carcinoma, mutant genotype of polymorphisms rs3218373 (OR = 2.38; 95% CI; 1.35–4.2; P = 0.0002), rs7802034 (OR = 4.21; 95% CI; 2.31–7.6; p = 0.0001) and rs3218536 (OR = 4.44; 95% CI; 2.54–7.7; P < 0.0001) were observed significantly associated with the said histological type. For pharyngeal carcinoma, mutant genotype of polymorphisms rs3218373 (OR = 2.47; 95% CI; 1.36–4.45; P = 0.002), rs7802034 (OR = 3.31; 95% CI; 1.73–6.35; P = 0.0003) and rs3218536 (OR = 2.65; 95% CI; 1.40–4.99; p = 0.002) were observed significantly associated with the pharyngeal carcinoma. For laryngeal carcinoma, mutant genotype of polymorphism rs3218373 (OR = 3.18; 95% CI = 1.64–6.16; P = 0.0006) and rs3218536 (OR = 5.04; 95% CI = 2.60–9.77; P < 0.0001) were observed significantly associated with said histological type of head and neck cancer, as shown in Table 4.

**Table 4:** Distribution of genotypes and odds ratios (OR) for different histological subtypes of HNC patients

Genotypes *XRCC2*	Controls (n = 400)	n	Oral cavity (n = 182) OR (95% CI)	P- value	n	Nasal Cavity (n = 88) OR (95% CI)	P- value	n	Pharynx (n = 78) OR (95% CI)	P- value	n	Larynx (n = 52) OR (95% CI)	P- value
**rs3218373**													
TT	299	104	1		30	1		41	1		22	1	
TG	52	30	0.94(0.57–1.54)	0.80	36	4.63(2.76–7.7)	<0.0001	17	1.86(1.01–3.43)	0.04	14	2.46(1.25–4.85)	0.009
GG	49	48	1.94(1.24–3.04)	0.003	22	2.38(1.35–4.2)	0.0002	20	2.47(1.36–4.45)	0.002	16	3.18(1.64–6.16)	0.0006
**rs2040639**													
AA	259	149	1		60	1		60	1		32	1	
AG	85	20	0.34(0.20–0.58)	<0.0001	16	0.82(0.45–1.4)	0.52	11	0.60(0.30–1.20)	0.15	13	1.23(0.63–2.41)	0.53
GG	56	13	0.31(0.16–0.59)	0.0004	12	0.96(0.49–1.8)	0.92	7	0.6090.26–1.38)	0.23	7	0.96(0.41–2.22)	0.91
**rs3218384**													
GG	269	159	1		65	1		63	1		44	1	
GC	79	21	0.39(0.23–0.66)	0.0005	13	0.70(0.37–1.3)	0.28	9	0.53(0.25–1.10)	0.09	5	0.43(1.16–1.12)	0.08
CC	52	2	0.07(0.01–0.30)	0.0003	10	0.85(0.41–1.7)	0.67	6	0.55(0.23–1.34)	0.19	3		
**rs7802034**													
AA	199	126	1		45	1		42	1		19	1	
AG	170	37	0.04(0.01–0.19)	<0.0001	20	0.39(0.23–0.6)	0.0008	19	0.43(0.25–0.75)	0.003	25	0.40(1.12–1.36)	0.14
GG	31	19	0.89(0.48–1.63)	0.70	23	4.21(2.31–7.6)	<0.0001	17	3.31(1.73–6.35)	0.0003	8	1.25(0.70–2.23)	0.44
**rs3218536**													
GG	336	129	1		44	1		48	1		19	1	
GA	26	20	1.13(0.61–2.09)	0.69	16	3.19(1.63–6.2)	0.0007	13	2.87(1.40–5.88)	0.003	15	5.83(2.83–11.9)	<0.0001
AA	38	33	1.93(1.16–3.22)	0.01	28	4.44(2.54–7.7)	<0.0001	17	2.65(1.40–4.99)	0.002	18	5.04(2.60–9.77)	<0.0001

OR, odds ratio; CI, confidence interval; p-value ≤ 0.05 considered as statistically significant. ORs were adjusted for age, sex and smoking status of study cohort in logistic regression model.

In case of genotype frequency correlation and smoking status of head and neck cancer, logistic regression model analysis was conducted using SNPs genotypes as a dependent variable and demographic parameters such as age, sex and smoking status as independent variable (Table 5). The results showed that smoking risk factor was associated with increased frequency of mutant genotype of rs3218373 (OR = 2.33; 95% CI = 1.003–5.42; P < 0.04) and rs3218536 (OR = 4.39; 95% CI = 1.007–19.10; P < 0.04) in head and neck cancer as shown in Table 5.

**Table 5:** Association of selected polymorphisms of *XRCC2* gene with smoking status.

Polymorphisms	B	S.E	Wald	Sig	OR	95% CI
rs3218373	0.826	0.428	3.720	0.04	2.33	1.003–5.42
rs2040639	−0.264	0.428	0.38	0.53	0.768	0.332–1.77
rs3218384	0.248	0.527	0.191	0.62	1.281	0.42–3.893
rs7802034	−0.606	0.562	1.164	0.281	0.545	0.181–1.641
rs3218536	1.478	0.751	3.877	0.04	4.386	1.007–19.10

Abbreviations: OR, odds ratio; CI, confidence interval; p-value ≤ 0.05 considered as statistically significant. ORs were adjusted for age and sex status of study cohort in logistic regression model.

### Haplotype analysis of the *XRCC2* SNPs

It was also investigated whether the five SNPs were in linkage disequilibrium. Any common haplotypes associated with the disease and rare haplotypes (with frequency < 5%) were excluded from the association analysis. The most common haplotypes of the five polymorphisms, calculated by Haploview 4.2, are summarized in Table 6.

**Table 6:** The distribution of *XRCC2* haplotypes in HNC patients and controls.

rs3218373	*XRCC2 haplotypes (SNPs)*			**rs3218536**	*Frequency*		*x²*	*P-value*
	**rs2040639**	**rs3218384**	**rs7802034**		**Cases**	**Controls**		
G	A	C	A	G	0.004	0.000	—	—
G	A	C	A	G	0.000	0.001	—	—
G	A	C	G	G	0.000	0.016	12.9	0.00
G	A	G	A	A	0.060	0.011	27.4	**1.6e**–**007**
G	A	G	A	G	0.139	0.048	39.9	**2.7e**–**010**
G	A	G	G	G	0.054	0.030	5.88	0.015
G	G	C	A	A	0.009	0.002	—	—
G	G	C	A	G	0.019	0.010	2.31	0.128
G	G	C	G	A	0.010	0.002	3.85	0.049
G	G	C	G	G	0.014	0.010	0.63	0.424
G	G	G	A	G	0.016	0.026	1.88	0.169
G	G	G	G	G	0.008	0.027	8.27	0.004
T	A	C	A	A	0.003	0.007	—	—
T	A	C	A	G	0.000	0.088	73.1	**1.2e**–**017**
T	A	C	G	A	0.000	0.006	—	—
T	A	C	G	G	0.000	0.057	46.9	**7.6e**–**012**
T	A	G	A	A	0.102	0.072	4.56	0.032
T	A	G	A	G	0.288	0.332	3.35	0.066
T	A	G	G	A	0.054	0.005	33.3	**8.1e**–**009**
T	A	G	G	G	0.087	0.081	0.22	0.637
T	G	C	A	A	0.014	0.002	6.18	0.012
T	G	C	A	G	0.025	0.009	5.83	0.015
T	G	C	G	A	0.011	0.002	4.38	0.036
T	G	C	G	G	0.003	0.010	3.53	0.060
T	G	G	A	A	0.000	0.012	8.02	0.004
T	G	G	A	G	0.010	0.095	57.2	**4.0e**–**014**
T	G	G	G	G	0.012	0.035	9.91	**0.001**
G	A	G	G	A	0.035	0.000	29.0	**7.2e**–**008**
G	G	G	A	A	0.016	0.000	13.3	0.000
T	G	G	G	A	0.005	0.000	—	—

Abbreviations: SNP, single nucleotide polymorphism; OR, odds ratio; CI, confidence interval; *p-value ≤ 0.05 considered statistical significant. P values in bold have still maintained their significance after Bonferroni correction.

**Figure 1:**
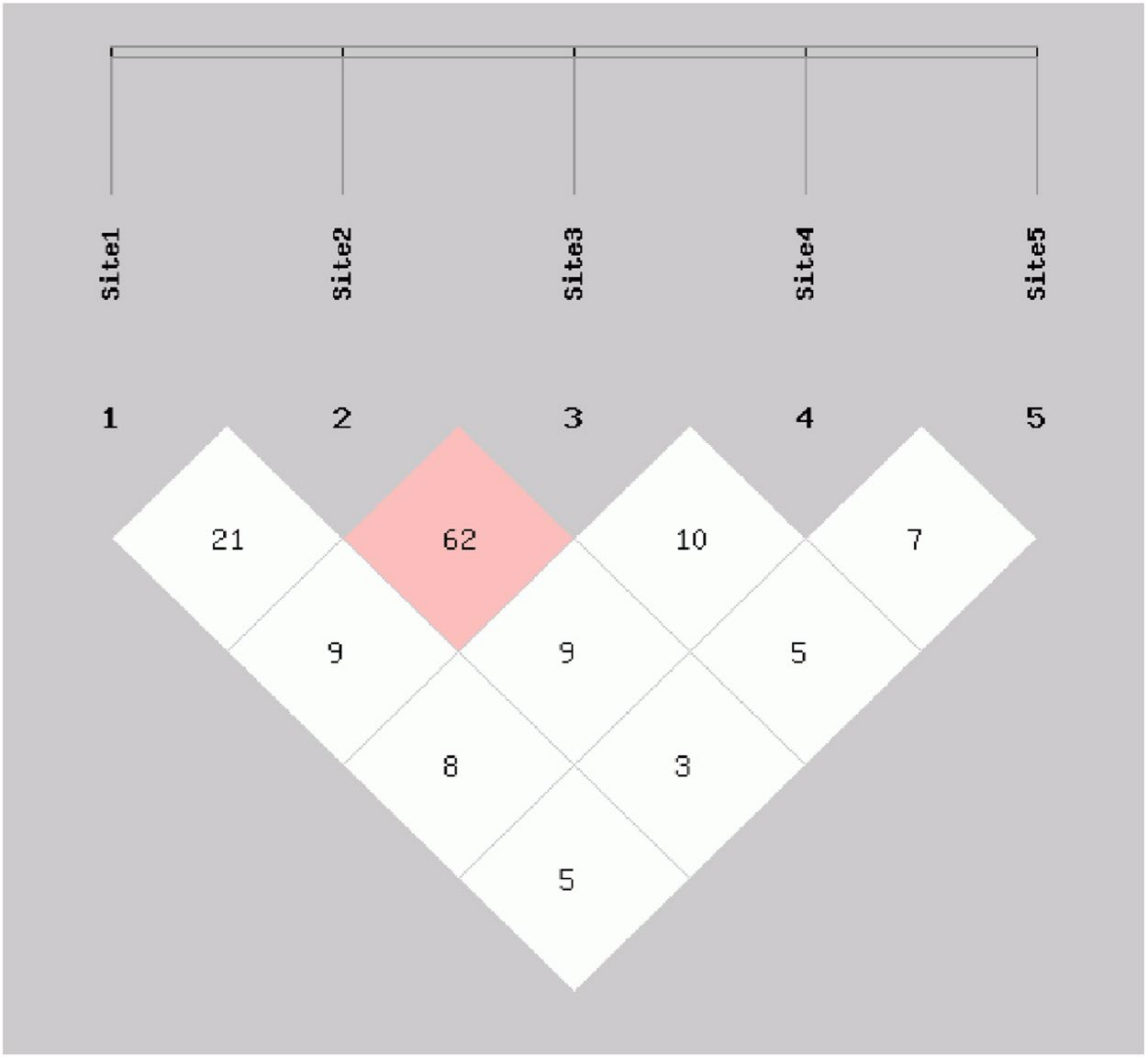
Pairwise linkage disequilibrium plot for examined *XRCC2* SNPs. Site 1 is for rs3218373, site 2 is for rs2040639, site 3 for rs3218384, site 4 for rs7802034 and site 5 for rs3218536. The darker region shows higher r^2^-value.

The haplotypes were generated using the five *XRCC2* intragenic SNPs (rs3218373, rs2040639, rs3218384, rs7802034 and rs3218536) among the HNC cases and controls, and thirty different haplotypes were generated which accounted for most of the haplotypes in the cancer patients and control groups (with frequency < 5%). For commonly observed haplotypes, GAGAA haplotype (P = 1.6e-007), GAGAG haplotype (P = 2.7e-010), GAGGG haplotype (P = 0.02), GGCGA haplotype (P = 0.04), TAGAA haplotype (P = 0.03), TAGGA haplotype (P = 8.10e-009), TGCAA haplotype (P = 0.01), TGCAG haplotype (P = 0.01) and TGCGA haplotype (P = 0.03) were found linked with significant increase in head and neck cancer risk while GGGGG (P = 0.0004), TGGAA (P = 0.004), TGGAG (P = 4.06e-014) and TGGGG (P = 0.001) were observed associated with a significant reduction in head and cancer risk. The other seventeen common haplotypes including GACAG, GACGA, GACGG, GGCAA, GGCAG, GGCGG, GGGAG, TACAA, TACAG, TACGA, TACGG, TAGAG, TAGGG,TGCGG, GAGGA, GGGAA and TGGGA were observed not associated with the risk of head and neck cancer as shown in Table 6. Since, this study was based on a relatively small sample size, we applied a Bonferroni correction to decrease the type I error. Following the Bonferroni correction the haplotypes GAGAA, GAGAG, TACAG, TACGG, TAGGA, TGGAG and GAGGA still maintained their significance. Furthermore, two of the SNPs in *XRCC2* (rs2040639 and rs3218384) were in strong LD (Fig. 1).

### Combined genotype analysis of *XRCC2* SNPs

Table 7 summarizes the association studies among the combined genotypes of the four SNPs and overall risk for head and neck cancer using conditional logistic regression model. The analysis revealed that rs3218373 and rs2040639 had a positive correlation with increased risk of HNC (OR = 2.89; 95% CI = 0.12–0.72; P < 0.001). A significant positive correlation was also observed between rs3218373 vs rs3218384 (OR = 2.44; 95% CI = 0.99–6.033; P < 0.05) and between rs2040639 vs rs3218536 (OR = 2.51; 95% CI = 0.99–6.031; P < 0.05) with increased risk of HNC as shown in Table 7.

**Table 7:** Logistic regression model of SNP-SNP interactions and HNC risk.

SNP-SNP interactions	B	S.E	Wald	Sig	OR	95% CI
rs3218373 vs rs2040639	1.241	0.462	7.216	**0.001**	2.89	0.117–0.715
rs3218373 vs rs3218384	0.895	0.460	3.775	0.05	2.447	0.992–6.033
rs3218373 vs rs7802034	−0.199	0.455	0.192	0.66	0.819	0.336–1.998
rs3218373 vs rs3218536	0.629	0.463	1.841	0.175	1.875	0.756–4.648
rs2040639 vs rs3218384	0.488	0.475	1.053	0.305	1.628	0.642–4.133
rs2040639 vs rs7802034	0.414	0.448	0.852	0.386	1.512	0.628–3.642
rs2040639 vs rs3218536	0.919	0.470	3.822	0.05	2.508	0.998–6.305
rs3218384 vs rs7802034	0.225	0.458	0.242	0.623	1.252	0.510–3.073
rs3218384 vs rs3218536	−0.470	0.463	1.033	0.310	0.625	0.252–1.548
rs7802034 vs rs3218536	0.699	0.452	2.393	0.122	2.013	0.830–4.882

Abbreviations: OR, odds ratio; CI, confidence interval; p-value ≤ 0.05 considered as statistically significant. ORs were adjusted for age, sex and smoking status of study cohort in logistic regression model.

## Discussion

Many earlier studies have reported that the genes involved in DNA repair and in the maintenance of genome integrity plays a crucial role in protection against mutations. Although single nucleotide polymorphisms have been identified in these DNA repair genes, such as *XRCC2*, but the influence of specific genetic variants on repair phenotype and cancer risk has not yet been identified^27–31^. Thus, an attempt was undertaken in this study to determine whether single nucleotide polymorphisms (SNPs) in *XRCC2* gene are associated with head and neck cancer. In this study, we successfully genotyped a total of five SNPs in different regions of *XRCC2* gene such as promoter region, exonic region and intronic region and examined their possible association with head and neck cancer risk. We also investigated that whether or not these five polymorphisms are in linkage disequilibrium and common haplotypes of these SNPs are associated with head and neck carcinogenesis. Finally, we estimated the association among the combined genotypes of five selected SNPs and overall risk of head and neck cancer.

The majority of earlier studies on cancer susceptibility have focused only on *XRCC2* rs3218536 SNP. In present study five polymorphisms, present in different regions of *XRCC2* gene, such as promoter region, exonic region and intronic region were screened in HNC cancer patients and controls. Among selected promoter polymorphisms, frequency of risk allele of first promoter polymorphism rs3218373 was observed significantly higher in patients compared to controls. However, in the case of other two promoter polymorphisms (rs2040639 and rs3218384), frequency of risk allele was observed significantly higher in controls compared to patients. Similar results have earlier been reported where significant association was observed between *XRCC2* promoter polymorphism and oral cancer risk^32^, breast cancer risk^33^, thyroid cancer risk^34^ and bladder cancer risk^35^. Although the functional consequences of these polymorphisms are unknown, their location in important domain(s) of *XRCC2* may control translation and mRNA decay and are also sites for RNA interference^34^.

In this study, fourth selected polymorphism of *XRCC2* gene, rs7802034, was located in the non-coding region and mutant allele frequency was observed associated with increased risk of HNC. Even though intronic SNPs are unlikely to have a direct functional role, still several studies have provided evidence that SNPs located in non-coding DNA, especially in intronic gene regions near the exon/intron boundaries, can inactivate pre-mRNA splice sites consequently affecting gene expression^36,37^, or can activate cryptic splice sites leading to exonization^38^. Furthermore, the presence of SNPs in 3‘-UTR of selected genes can modify the binding with specific microRNAs (miRNAs)^39^. For fifth selected polymorphism of *XRCC2* gene, rs3218536, frequency of risk allele was observed significantly higher in patients compared to controls. Similar results have earlier been reported where significant association was observed between *XRCC2* polymorphism rs3218536 with laryngeal and pharyngeal cancer risk^40^, thyroid cancer risk^41^, ovarian cancer^42^, gastric cancer risk^43^, oral cancer^44^ and head and neck cancer^45^. Nevertheless, some of the studies have also reported that there is no significant association between *XRCC2*- rs3218536 polymorphism and thyroid cancer^46,47^. In some of the studies rs3218536 polymorphism has been considered a genetic adjuster for ovarian and colorectal cancer patients^48,49^. The influence of these specific genetic variants on repair phenotype and cancer risk is yet not clear. However, amino acid 188 is conserved in humans, mice and rat *XRCC2* proteins as well as human RAD51C, suggesting a potential functional role in DNA repair activity^50^. Romanowicz-Makowska *et al*., (2016) reported that rs3218536 polymorphism has shown a functional significance and may be responsible for a low DNA repair capacity phenotype characteristic of cancer patients including larynx carcinoma^31^. Future determination of functional and active sites in human *XRCC2* protein may clarify the biological importance of this amino acid residue.

It is believed that haplotype analysis can provide more information than single-locus analysis^19^. In second step of study, we successfully established haplotypes for the *XRCC2* gene from different combinations of five SNPs. GAGAA, GAGAG, GAGGG, GGCGA, TAGAA, TAGGA, TGCAA, TGCAG and TGCGA haplotypes of selected polymorphism were observed linked with significant increase in head and neck cancer risk. These haplotypes possess risky alleles which are consistently over represented in head and neck cancer patients relative to healthy controls, suggesting a leading role of selected polymorphisms in risk determination. To produce more information, linkage disequilibrium was calculated for five SNPs of *XRCC2* gene and rs2040639 was found in complete linkage disequilibrium with rs3218384. It is possible that SNPs of this gene may have a collective effect on DNA repair outcomes. It has earlier been reported that interaction of polymorphisms, of the same gene or other genes, by linkage disequilibrium may be important in modulating the overall repair activity. This might explain the influence of genetic variations in the carcinogenic process^51^.

After this step, SNP-SNP interaction was calculated which showed that rs3218373-rs2040639 and rs2040639-rs3218536 combinations were associated with an increased head and neck cancer risk. Similar results have earlier been reported where it was suggested that mutations in *XRCC2* gene may contribute to decreased or lost DNA repair capacity^12,46^. Furthermore, SNPs of *XRCC2* may also increase the risk of several types of cancer including thyroid, brain and breast cancer^52,53^. In previously reported studies, cumulative meta-analyses have suggested no such significant association. Theoretically, genetic variants in *XRCC2* gene can change the regular function of this gene, disturb the DNA repair and subsequently increase the cancer risk^54^. Nevertheless, some of the previous studies have reported that the variant alleles of this polymorphism can increase resistance to DNA damage induced by cisplatin^50,55^ which enlightens protective function of this polymorphism under certain conditions.

In conclusion, current evidence suggest that analyzed *XRCC2* polymorphisms are directly associated with HNC risk. Nevertheless, several potential limitations of this study need to be considered before making a final conclusion. Firstly, studies should incorporate a larger sample size with various ethnic groups to further confirm the association between SNPs of *XRCC2* and head and neck susceptibility. Secondly, HNC is a polygenic disease, therefore other genetic and environmental factors should also be assessed. Thirdly, few well-known risk factors for HNC such as human papilloma virus (HPV) has been discovered. In such cases correlation between sexual behavior of the subjects and head and neck carcinogenesis should be considered. Fourthly, subjects in this case-control study came from two hospitals and this may cause selection bias that can have substantial impact on the overall conclusions. As a result, large-scale studies adjusting for a wide range of factors are recommended to validate these findings. In conclusion, our results indicate that these five polymorphisms of *XRCC2* gene may be related to individual susceptibility to head and neck risk in Pakistani population.

## Electronic supplementary material

supplementary Tables
