# Supplementary material for: Haplotype analysis of XRCC2 gene polymorphisms and association with increased risk of head and neck cancer
Source: Sci Rep. 2017 Oct 16;7:13210. doi: 10.1038/s41598-017-13461-6 (PMC5643489; doi:10.1038/s41598-017-13461-6)
Supplement: Supplementary file 1 — supplementary Tables [file 41598_2017_13461_MOESM1_ESM.pdf]

Haplotype analysis of XRCC2 gene polymorphisms and association with increased risk of head and neck cancer

Soma Saeed, Ishrat Mahjabeen, Romana Sarwar, Kashif Bashir, Mahmood Akhtar Kayani*

Supplementary Table S1: Primer sequences of five SNPs of XRCC2 gene (rs3218373, rs2040639, rs3218384, rs7802034 and rs3218536).

	Sequence (5' - 3')	*Product size (bp)*	*Annealing temp.(ºC)*
Gene XRCC2			
rs3218373			
Wild forward	TAAAGCCCATTTGTTTCAAG	137bp	54
Mutant forward	TAAAGCCCATTTGTTTCAAT		
Common reverse	AAACGCTAGGAAAGAGCATA		
rs2040639			
Wild reverse	GCACACCTGTTCGTGTGACT	225bp	**60**
Mutant reverse	GCACACCTGTTCGTGTGACC		
Common forward	GTTGTAAACCAGCCTAGGCAAC		
rs3218384			
Wild reverse	ACTCTACGGCCAGTCAAATG	311bp	**58**
Mutant reverse	ACTCTACGGCCAGTCAAATC		
Common forward	GCCTGCTTGTGCAATACAATTA		
rs3218536			
Wild reverse	CGTTGCAAAAAGAACCAGTC	227bp	**56**
Mutant reverse	CGTTGCAAAAAGAACCAGTT		
Common forward	ACTGCAGTAGTAGCACCCAC		
rs7802034			
Wild reverse	CTCCTTACAGCGTTCTATGT	220bp	**60**
Mutant reverse	CTCCTTACAGCGTTCTATGC		
Common forward	GTCCTACAGAAGGGACATCA		
Beta-Actin			
Forward	CGAGAAGATGACCCAGGTGA	496bp	**55**
Reverse	TACATGGCTGGGGTGTTGAA		

Supplementary Table S2: Frequency distribution analysis of selected SNPs on the basis of hospitals.

Genotype / Allele	*PIMS Hospital Cases n (%)*	*NORI Hospital Cases n (%)*	*OR (95% CI)*	*P- value*
rs3218373				
TT	91 (46.2%)	106 (53.8%)	0.73(0.49 to 1.09)	P = 0.1311
TG	45 (46.4%)	52 (53.6%)	0.74(0.42 to 1.31)	P = 0.3153
GG	48 (45.3%)	58 (54.7%)	0.68(0.39 to 1.17)	P = 0.1702
rs2040639				
AA	148 (49.2%)	153 (50.8%)	0.93(0.67 to 1.2)	P = 0.6836
AG	28 (46.7%)	32 (53.3%)	0.76(0.37 to 1.56)	P = 0.4655
GG	18 (46.2%)	21 (53.8%)	0.73(0.30 to 1.78)	P = 0.4973
rs3218384				
GG	162 (48.9%)	169 (51.1%)	0.91(0.67 to 1.24)	P = 0.5864
GC	20 (41.7%)	28 (58.3%)	0.51(0.22 to 1.14)	P = 0.1041
CC	10 (47.6%)	11 (52.4%)	0.82(0.24 to 2.77)	P = 0.7577
rs7802034				
AA	112 (48.3%)	120 (51.7%)	0.87(0.60 to 1.25)	P = 0.4577
AG	48 (47.5%)	53 (52.5%)	0.82(0.47 to 1.42)	P = 0.4819
GG	30 (44.8%)	37 (55.2%)	0.65(0.33 to 1.29)	P = 0.2274
rs3218536				
GG	110 (45.8%)	130 (54.2%)	0.71(0.5 to 1.02)	P = 0.0682
GA	30 (46.9%)	34 (53.1%)	0.77(0.38 to 1.55)	P = 0.4798
AA	45 (46.9%)	51 (53.1%)	0.77(0.44 to 1.37)	P = 0.3868

Abbreviations: N, number of samples; OR, odds ratio; CI, confidence interval;* p-value calculated by χ^2 -test.
